# Radiation combines with immune checkpoint blockade to enhance T cell priming in a murine model of poorly immunogenic pancreatic cancer

**DOI:** 10.1098/rsob.210245

**Published:** 2021-11-17

**Authors:** Courtney T. Stump, Kevin Roehle, Nataly Manjarrez Orduno, Stephanie K. Dougan

**Affiliations:** ^1^ Cancer Immunology and Virology, Dana-Farber Cancer Institute, Boston, MA 02215, USA; ^2^ Department of Gastroenterology, Massachusetts General Hospital, Boston, MA 02215, USA; ^3^ Department of Immunology, Harvard Medical School, Boston, MA 02215, USA; ^4^ Bristol Myers Squibb, Cambridge, MA 02142, USA

**Keywords:** immune checkpoint blockade, abscopal‌, radiation, pancreatic ductal adenocarcinoma, pancreatic cancer, immunotherapy

## Abstract

Radiation has been a pillar of cancer therapy for decades. The effects of radiation on the anti-tumour immune response are variable across studies and have not been explicitly defined in poorly immunogenic tumour types. Here, we employed combination checkpoint blockade immunotherapy with stereotactic body radiation therapy and examined the effect on tumour growth and immune infiltrates in subcutaneous and orthotopic mouse models of pancreatic cancer. Although immune checkpoint blockade and radiation were ineffective alone, their combination produced a modest growth delay in both irradiated and non-irradiated tumours that corresponded with significant increases in CD8+ T cells, CD4+ T cells and tumour-specific T cells as identified by IFNγ ELISpot. We conclude that radiation enhances priming of tumour-specific T cells in poorly immunogenic tumours and that the frequency of these T cells can be further increased by combination with immune checkpoint blockade.

## Graphical abstract

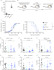

## 1. Background

Pancreatic malignancies remain among the deadliest cancers, with an overall survival rate under 10% [1]. Though survival varies based on cancer stage and type, 85% of pancreatic cancer patients are diagnosed with pancreatic ductal adenocarcinoma (PDAC) and are diagnosed post-metastatic spread [2]. Pancreatic tumours induce systemic immune and metabolic alterations, resulting in adipose and muscle wasting with significant morbidity [3,4]. Furthermore, PDAC is characterized by dense fibrotic stroma consisting of fibroblasts, extracellular matrix, macrophages, granulocytes and monocytes, which collectively form an immunosuppressive microenvironment [2,5]. Although both CD4+ and CD8+ T cells are present in pancreatic cancer, they are frequently located away from the tumour cell nests. Instead, tumour cells are more commonly found in close proximity to alternatively activated macrophages or fibroblasts, both of which portend poor prognosis [6–9].

Although combination chemotherapy regimens are the mainstay of pancreatic cancer treatment, radiation is frequently included, particularly in resectable or locally advanced disease as a means of reducing tumour burden prior to surgery [10–12]. Despite its widespread use, the clinical benefit of radiation in local or metastatic PDAC has not been established [11,13,14]. Accordingly, a variety of strategies have been used to increase the efficacy of radiation, including employing intraoperative radiotherapy, examining the unintentional targets of radiation, combining radiation with other therapies and using stereotactic beam radiotherapy (SBRT) following external beam radiotherapy (EBRT) [14–19].

Immunotherapies have transformed the state of cancer therapy [20,21]. While a variety of immunologic strategies have been employed against different tumours, the most successful has been immune checkpoint blockade therapy [22]. Monoclonal antibodies targeting programmed cell death protein-1 (PD-1) and cytotoxic T-lymphocyte-associated antigen 4 (CTLA-4) revolutionized the field by demonstrating that reinvigorating exhausted T cells in cancer patients was not only a viable strategy, but also was capable of inducing durable remissions in a subset of patients with diverse malignancies [22]. However, despite the successes of immune checkpoint blockade therapies in melanoma and other cancers, these treatments have largely proven ineffective against pancreatic tumours outside of the approximately 1% of PDAC tumours with deficiencies in mismatch repair genes [23–26].

Immune checkpoint blockade reinvigorates existing T cell responses and is thus most effective when used in patients with robust endogenous T cell priming. In order to increase the effectiveness of immune checkpoint blockade against pancreatic cancer, various strategies have been employed to augment priming and infiltration of tumour-specific T cells [27,28]. Notably, agonistic antibodies to CD40 have shown efficacy in early-stage trials of pancreatic cancer, in part via their ability to mobilize dendritic cells to present tumour antigens and prime naive T cells [29–32]. Cytotoxic therapies that lead to inflammatory tumour cell death can also release endogenous adjuvants from dying tumour cells, activating dendritic cells and enhancing T cell priming [33,34]. This concept is broadly termed immunogenic cell death, although the exact therapies responsible for inducing immunogenic cell death and the nature of released adjuvants remain unclear [35]. Regardless, radiation probably induces immunogenic cell death, and this stimulation of tumour-specific T cell responses can lead to the clinical phenomenon of shrinking tumour lesions outside the field of radiation [36]. These so-called abscopal responses are rare, indicating that the immunogenic effects of radiation are subtle and largely insufficient to induce robust T cell priming and tumour regressions. However, the combination of radiation with checkpoint blockade has shown clinical tolerability and even benefit in several tumour types, including melanoma, small cell lung cancer and renal cell cancer; understanding the mechanism of each agent will be critical to deciphering which patients or which tumour types might be most amenable to this approach [37–40].

To this end, a landmark study examined the immunologic effects of SBRT, anti-CTLA-4 and anti-PD-1 in mouse models of melanoma and pancreatic cancer, and found that radiation plays a non-redundant role in combination with CTLA-4 and PD-1 blockade [41]. The authors used flow cytometric analysis of immune infiltrates to demonstrate that as part of combination therapy CTLA-4 blockade reduces activity of regulatory T cells, whereas PD-1 blockade increases proliferation and activation of CD8+ T cells. Radiation did not augment T cell frequencies but, importantly, broadened the clonality of the T cell response as indicated by sequencing of the TCR genes [41]. Radiation and PD-1 blockade are also effective in mice with orthotopically implanted PDAC tumours that show baseline responsiveness to single-agent PD-1 blockade, indicating that radiation can further enhance T cell responses to immunogenic tumours [42]. Combination of radiation and agonistic anti-CD40 in mouse models of pancreatic cancer also increases T cell priming, as evidenced by the development of local vitiligo from loss of tolerance to melanocyte antigens in the irradiated field [43,44].

Here, we used a poorly immunogenic model of pancreatic cancer that is critically unresponsive to combination checkpoint blockade. We investigated the immunologic impact of combining dual CTLA-4 and PD-1 blockade with radiation and compared tumour immune infiltrates and therapeutic efficacy in the subcutaneous and orthotopic settings. We show only PDAC immune infiltrates in a distant, unirradiated tumour, indicating the combined systemic effects of radiation and immune checkpoint therapy. We find that addition of SBRT extended mouse survival and correlated with increased IFNγ-producing, tumour-specific T cells. These results support the use of radiation to induce immunogenic cell death as part of a combination immunotherapy regimen, even in cancers that are otherwise poorly immunogenic.

## 2. Methods

### 2.1. Cell line

The 6694c2 cell line was derived from a LSL-KrasG12D;p53+/floxed, Pdx-cre, YFP-floxed mouse and was a gift from Ben Stanger (University of Pennsylvania) [45]. Cells were cultured at 37°C with 5% CO_2_ in RPMI media (Life Technologies) supplemented with 10% (v/v) FBS, 2 mmol l^−1^ L-glutamine (Gibco), 1% (v/v) penicillin/streptomycin (Gibco), 1% (v/v) MEM non-essential amino acids (Gibco), 1 mmol l^−1^ sodium pyruvate (Gibco) and 0.1 mmol l^−1^ β-mercaptoethanol (Sigma).

### 2.2. Animal models

C57BL/6 mice were purchased from Jackson Laboratories (stock no. 000664) and housed according to DFCI guidelines.

### 2.3. Subcutaneous tumours

A total of 200 000 6694c2 cells were subcutaneously injected in HBSS (Gibco). Tumour size was measured twice weekly, and tumour volume calculated by multiplying the three dimensions of the tumour. Mice reached study endpoint criteria and were euthanized with CO_2_ when tumours reached 2000 mm^3^ or had gross ulceration. Other humane endpoint criteria included body condition score less than or equal to 2 or weight loss greater than 10% of initial body weight, although no mice in this study met these humane endpoints.

### 2.4. Orthotopic tumours

Orthotopic tumours were inoculated as previously described [46]. Briefly, C57BL/6 mice were anaesthetized with a ketamine/xylazine cocktail, shaved on the left flank and the surgical site cleaned with ethanol and betadine. An incision was made in the skin and peritoneum, and the pancreas externalized with forceps. 6694c2 cells were resuspended in phosphate-buffered saline (PBS) and mixed 1 : 1 by volume with matrigel (Corning) for a total of 100 000 cells per 30 µl. The cell suspension was kept on ice and drawn into a chilled insulin syringe. Cells were then injected into the tail of the pancreas, and a bubble was observed. Mice that showed signs of leakage were removed from the experiment. The pancreas was left external to the body cavity for 1 min with the mice on a warming pad to solidify the matrigel. The pancreas was then reinserted, peritoneum sutured with one stitch of absorbable suture and the skin stapled with a sterile wound clip. Mice were given analgesia (Buprenex) and monitored post-surgery according to protocols approved by the Dana-Farber IACUC. Mice were euthanized no more than 18 days post-surgery. Tumours were weighed at the time of euthanasia.

### 2.5. *In vivo* treatment

Mice treated with immune checkpoint blockade were administered 10 mg kg^−1^ αPD-1 (BMS RMP1-14), and/or 10 mg kg^−1^ αCTLA-4 (BMS 9H10) weekly. Control mice were treated with 10 mg kg^−1^ of each relevant isotype control, also weekly. Treatment was begun 6 days after tumour implantation.

### 2.6. Radiation

Mice were inoculated with bilateral subcutaneous flank tumours (described above), and one of the two tumours was irradiated with a single 5 Gy dose of SBRT. A small animal radiation research platform was used for radiation, allowing radiation to be delivered precisely to the tumour site.

### 2.7. Flow cytometry

Tumours were excised, and orthotopic tumours were weighed. Tumours were chopped and incubated at 37°C in RPMI with collagenase IV (Sigma) and trypsin inhibitor (Life Technologies). Tumour pieces were further manually digested before being filtered through a 40 μM cell strainer, washed with PBS and centrifuged. The resulting pellet was resuspended in PBS with 2% FBS and stained with two separate panels of flow cytometry antibodies. Samples were stained for 30 min at 4°C, washed with PBS and resuspended in 1% formalin before analysis on a Sony spectral flow cytometer (SP6800). Flow cytometry antibodies were purchased from BioLegend. Myeloid panel: CD11c FITC (no. 117305), CX3CR1 PE (no. 149006), Ly6G PE-Cy7 (no. 127617), SiglecF Brilliant Violet 421 (no. 155509), CD11b Pacific Blue (no. 101223), Ly6C Brilliant Violet 570 (no. 128030), I-A/I-E Brilliant Violet 510 (no. 107635), CD45 Brilliant Violet 711 (no. 103147), NK1.1 Brilliant Violet 785 (no. 108749), F4/80 APC (no. 123116).

Lymphoid panel: CD45 Brilliant Violet 711 (no. 103147), CD11b Pacific Blue (no. 101223), CD25 Alexa Fluor 488 (no. 102017), CD103 PE (no. 121405), PD-1 PE-Cy7 (no. 109109), CD4 Brilliant Violet 510 (no. 100553), B220 Brilliant Violet 605 (no. 103243), CD8α Brilliant Violet 785 (no. 100749), CD11c APC (no. 117309).

### 2.8. ELISpot

ELISpot plate (BD Biosciences) was treated with sterile-filtered 70% ethanol before being washed three times with sterile PBS 1×. IFNγ capture antibody (BD Biosciences no. 551881) was plated and the plate was sealed and left to incubate at 4°C overnight. Positive control wells were also plated with anti-CD3ε (BioLegend no. 100340). The plate was washed three times with sterile PBS and blocked with 10% (v/v) FBS in PBS overnight at 4°C. IFNγ-stimulated 6694c2 or B16F10 cells were plated, except in the unstimulated and positive control wells. Pancreatic draining lymph nodes were excised from treated mice bearing orthotopic tumours, and lymph nodes were macerated to form a single-cell suspension. Lymph node cells (one-third of lymph node per well) were plated on top of pre-plated tumour cells with human IL-2 (PeproTech), and positive control wells also received anti-CD28 (BioLegend no. 102116). Plate was incubated at 37°C for 24 h before being washed with sterile water followed by PBST. IFNγ detection antibody (BD Biosciences no. 551881) was added, and plate was incubated for 2 h at room temperature. Wells were washed with PBST, and streptavidin-HRP (BD Biosciences) was added. Plate was incubated for 1 h at room temperature before being washed with PBST and PBS. AEC chromagen substrate (BD Biosciences) was added, and plate was developed. Plate was dried and analysed.

## 3. Results

### 3.1. Pancreatic cancer is poorly immunogenic

**Figure 1.**
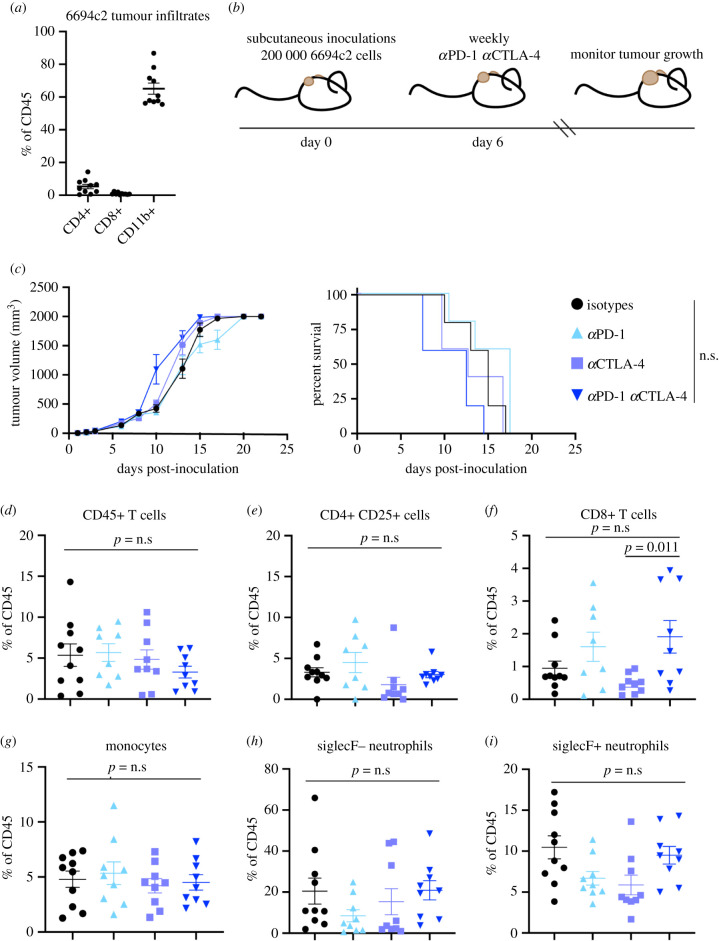
Checkpoint blockade treatment in the absence of radiation is ineffective against a poorly immunogenic pancreatic cancer cell line. (*a*) C57BL/6 mice were inoculated with bilateral subcutaneous 6694c2 tumours. Tumours were harvested at day 16, digested and stained for myeloid and lymphoid markers, and then analysed by spectral flow cytometry. (*b*) Schematic of experimental timeline for tumour growth and survival data. (*c*) C57BL/6 mice were implanted with subcutaneous 6694c2 tumours and treated intraperitoneally with isotype controls, anti-PD-1, anti-CTLA-4 or combination anti-PD-1 and anti-CTLA-4 (200 µg each antibody) every 7 days starting on day 6. Tumour size and survival were monitored. *n* = 5 mice per group. *p-*values were calculated by Mantel–Cox log rank test. (*d*–*i*) C57BL/6 mice were inoculated with bilateral subcutaneous 6694c2 tumours. Mice were administered 200 µg each of anti-PD-1, anti-CTLA-4 or isotype controls intraperitoneally every 7 days starting on day 6. Tumours were harvested at day 16, digested and stained for myeloid and lymphoid markers, and then analysed by spectral flow cytometry. *n* = 5 mice in the isotype group and *n* = 4 mice per group in the other groups. Data are mean ± s.e.m. Data points show biological replicates. *p-*values were determined by ANOVA.

To characterize the immune microenvironment of 6694c2 pancreatic tumours, we implanted subcutaneous tumours into immune-competent C57BL/6 mice and assessed tumour immune infiltrates by flow cytometry. Tumour immune infiltrates were primarily comprised CD11b+ myeloid cells, with a paucity of CD4+ and CD8+ T cells, as previously reported (figure 1*a*) [45]. We then assessed the response of 6694c2 tumours to immune checkpoint blockade. Anti-PD-1 and anti-CTLA-4, delivered alone or in combination, did not induce tumour regression or improve mouse survival (figure 1*b*,*c*).

Although immune checkpoint blockade therapy did not affect tumour growth, it could still have altered immune populations within the tumour microenvironment. Accordingly, we harvested subcutaneous tumours from mice treated with anti-PD-1, anti-CTLA-4, both anti-PD-1 and anti-CTLA-4 or relevant isotype controls, and we assessed immune infiltrates by flow cytometry (electronic supplementary material, figure S1). There were no significant changes in intratumoural frequencies of CD4+ T cells, including CD4+ CD25+ cells, which include but are not necessarily entirely composed of Tregs, among any of the groups (figures 1*d*,*e*). Tumour-infiltrating CD8+ T cell frequencies were mostly unaffected, although intratumoural CD8+ T cell frequencies increased between mice treated with single-agent anti-CTLA-4 and mice treated with combination checkpoint blockade (figure 1*f*). Frequencies of various myeloid populations in the tumour, including monocytes and neutrophils, were not affected by therapy (figures 1*g*–*i*).

### 3.2. Radiation improves the anti-tumour response to immune checkpoint blockade

**Figure 2.**
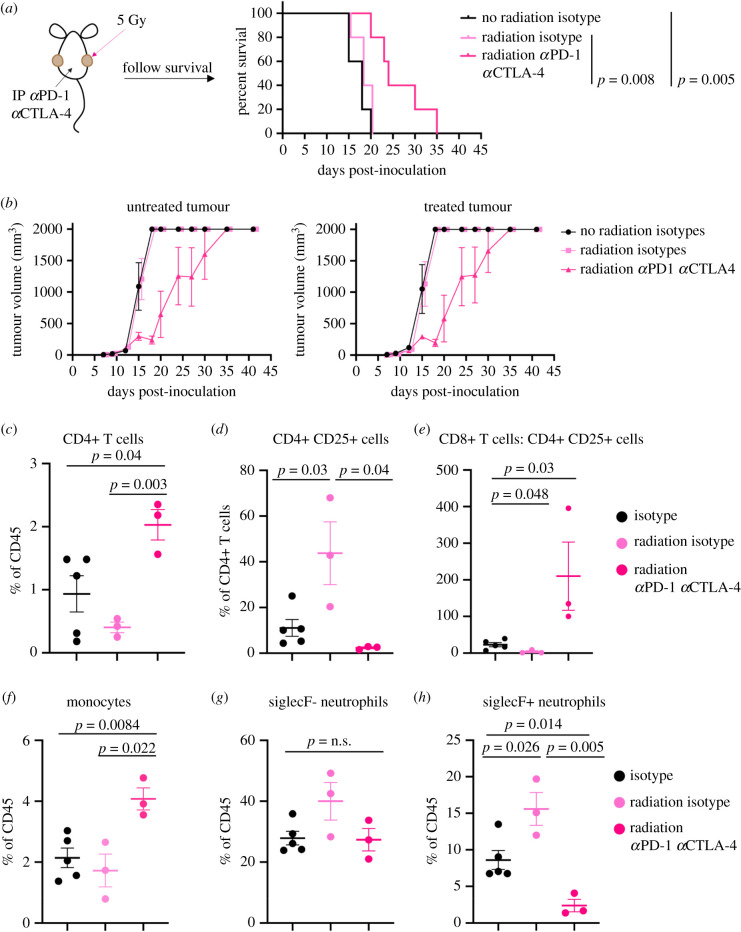
Checkpoint blockade and radiation synergistically promote anti-tumour immunity in a subcutaneous model of pancreatic cancer. (*a*) C57BL/6 mice were inoculated with bilateral subcutaneous 6694c2 tumours. Six days following tumour inoculation, one of the two tumours was irradiated with one dose of 5 Gy, while the other tumour remained untreated in order to allow potential evaluation of the abscopal effect. Mice were administered 200 µg each of anti-PD-1, anti-CTLA-4 or isotype controls intraperitoneally every 7 days starting on day 6. Survival was monitored. *n* = 5 mice per group. *p-*values were calculated by Mantel–Cox rank log test. (*b*) Tumour growth curves for the experiments described in (*a*). Results are representative of two independent experiments. (*c*–*h*) Mice were implanted with bilateral subcutaneous 6694c2 tumours, and one tumour was irradiated with 5 Gy on day 6 after tumour inoculation. Mice were administered 200 µg each of anti-PD-1, anti-CTLA-4 or isotype controls intraperitoneally every 7 days starting on day 6. At day 15 post-inoculation, tumours were harvested, digested, and stained for flow cytometric analysis. Only immune infiltrates from the non-irradiated tumour shown. *n* = 5 in the isotype alone group, and *n* = 3 mice in the radiation isotype and radiation αPD-1 αCTLA-4 groups. Group size is smaller in radiation isotype and radiation αPD-1 αCTLA-4 groups because of premature tumour ulceration. Graphs show mean ± s.e.m. *p-*values were determined by ANOVA.

We next wondered whether radiation, delivered in addition to combination checkpoint blockade therapy, would enhance the minimal effect of the checkpoint blockade delivered alone. Mice were inoculated with bilateral subcutaneous 6694c2 tumours, one of which was irradiated once with 5 Gy 6 days post-tumour inoculation. Combination anti-PD-1 and anti-CTLA-4 therapy was also initiated on the day of radiation (figure 2*a*). Radiation alone had no effect on tumour growth of either the irradiated or non-irradiated side, consistent with the radioresistant nature of PDAC as previously reported [44]. Combination of radiation and checkpoint blockade therapy moderately delayed tumour growth and extended survival (figure 2*a*,*b*). Both the treated and untreated tumours of mice receiving combination therapy grew at slower rates, suggestive of a systemic anti-tumour immune response.

To understand the immunological underpinnings of systemic tumour control provided by combination radiation and immune checkpoint blockade therapy, we harvested non-irradiated tumours at a mid-point of growth and assessed immune infiltrates. Tumour-infiltrating effector CD4+ T cells increased in combination-treated mice, compared to both isotype-treated and radiation-treated mice (figure 2*c*). CD4+ CD25+ T cells increased in the untreated tumours of mice treated with radiation alone; however, this increase in intratumoural Tregs was abrogated in the combination-treated mice, leading to an overall significant increase in the intratumoural CD8+ T cell-to-Treg ratio in mice treated with the combination therapy (figures 2*d*,*e*).

Previous reports of radiation in mouse models of pancreatic cancer revealed an increase in immunosuppressive monocytes recruited by CCL2, and combination therapy modestly increased the frequency of tumour-infiltrating monocytes (figure 2*f*) [47]. We did not observe significant alterations in the frequency of conventional neutrophils, all given limitations in sample size. Immunosuppressive SiglecF+ neutrophils were increased in mice treated with radiation alone, and importantly, SiglecF+ immunosuppressive neutrophils were significantly decreased by the combination of radiation and immune checkpoint blockade (figure 2*h*).

### 3.3. The immune microenvironment differs between subcutaneous and orthotopic pancreatic tumours

**Figure 3.**
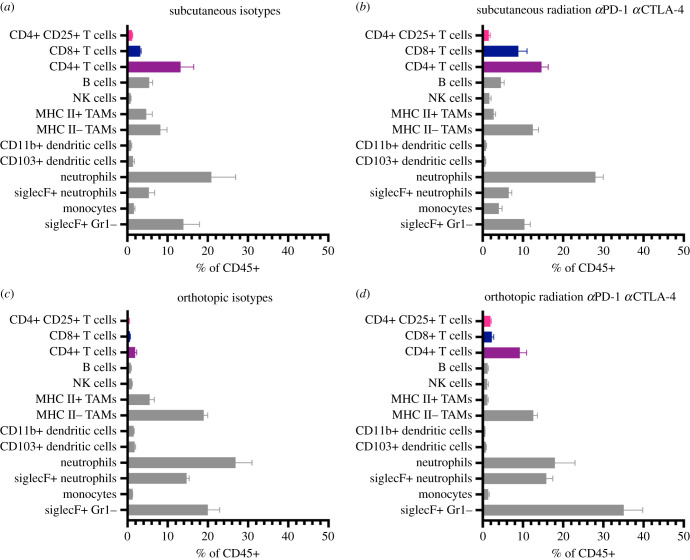
Orthotopic pancreatic tumours contain fewer T cells than subcutaneous tumours, before and after treatment. (*a*–*d*) C57BL/6 mice were inoculated with either bilateral subcutaneous 6694c2 tumours or one subcutaneous tumour and one orthotopic tumour. Six days following tumour inoculation, the right flank subcutaneous tumour was irradiated with one dose of 5 Gy, while the other tumour remained untreated. Mice were administered 200 µg each of anti-PD-1, anti-CTLA-4 or isotype controls intraperitoneally every 7 days starting on day 6. Tumours were harvested at day 15 post-inoculation and the non-irradiated tumour was digested, stained and analysed by spectral flow cytometry. Pie charts show mean values of tumour-infiltrating immune populations as per cent of CD45+ normalized to the sum of all identified CD45+ cells. *n* = 5 mice per group.

To determine whether radiation and combination checkpoint blockade would affect the immune microenvironment of primary pancreatic tumours, we compared the immune composition of 6694c2 tumours implanted either subcutaneously or orthotopically in the pancreas. In both cases, mice were also inoculated with a subcutaneous tumour on the right flank to serve as the irradiated lesion. We compared the immune microenvironment of the non-irradiated orthotopic or subcutaneous tumours (figure 3*a–d*) in order to understand any potential abscopal effect of the therapy. Orthotopic pancreatic tumours had fewer CD4+ and CD8+ T cell infiltrates than subcutaneous tumours and a larger fraction of SiglecF+ granulocytes. Combination radiation and immune checkpoint blockade modestly shifted the immune profile of orthotopic tumours but did not increase CD4+ or CD8+ T cells to the frequencies observed in subcutaneous tumours (figure 3*a*–*d*).

### 3.4. Radiation and checkpoint blockade augment T cell priming against pancreatic cancer in the orthotopic setting

**Figure 4.**
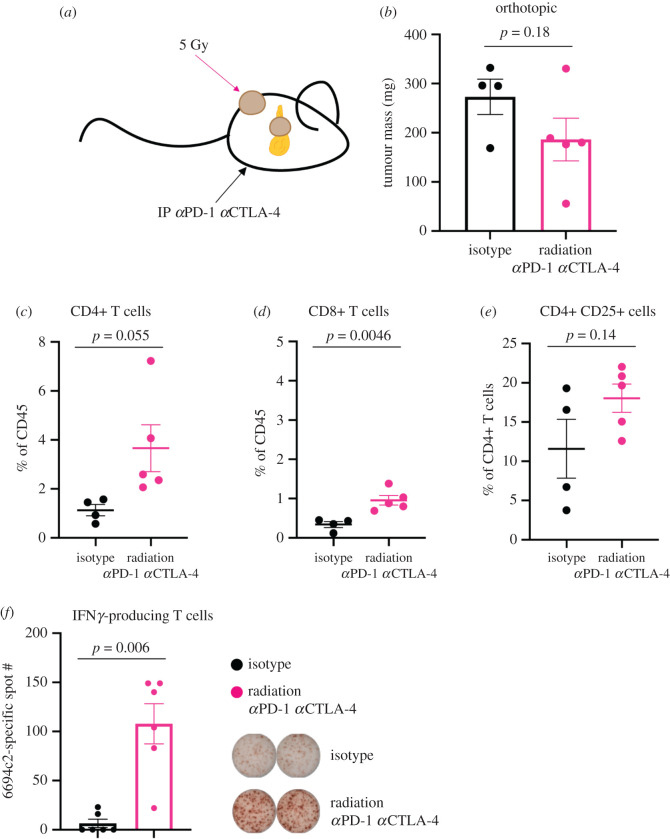
Systemic anti-PD-1 and anti-CTLA-4, combined with radiation at a distant tumour site, induces immune-mediated tumour control in the orthotopic setting. (*a*) Diagram showing that radiation was delivered to the subcutaneous tumour only. (*b*–*e*) Mice were implanted with subcutaneous and orthotopic 6694c2 tumours. On day 6 following inoculation, the subcutaneous tumour was irradiated (1 × 5 Gy). Mice were administered 200 µg each of anti-PD-1, anti-CTLA-4 or isotype controls intraperitoneally every 7 days starting on day 6. At day 14, orthotopic tumours were harvested, weighed and digested. Following tumour digestion, cells were stained for immune markers for spectral flow cytometry analysis. (*f*) In addition to harvesting orthotopic tumours, pancreatic draining lymph nodes were harvested from orthotopic tumour-bearing mice. Lymph nodes were mechanically digested, and cells were cocultured with 6694c2 cells or an unrelated cell line and analysed for IFNγ production by ELISpot. Spot number is reported with background subtracted. Data are shown as mean ± s.e.m. and *p-*values were calculated by Mann–Whitney test. *n* = 5 mice in the radiation αPD-1 αCTLA-4-treated group and *n* = 4 mice in the isotype-treated group; one mouse reached endpoint in the study prior to tumour harvest.

To more closely determine the effect of radiation and combination checkpoint blockade on orthotopic tumour growth, rather than to compare subcutaneous and orthotopic tumours, we followed the same treatment scheme, whereby mice were inoculated with both a subcutaneous tumour (to serve as the irradiated lesion) and an orthotopic tumour, and mice were treated as shown (figure 4*a*). Combination radiation and immune checkpoint blockade induced a trending decrease in orthotopic tumour mass in treated mice that did not reach statistical significance, potentially due to limitations in sample size (figure 4*b*).

To determine whether combination treatment affected the T cell response to orthotopic PDAC tumours, we analysed immune infiltrates by flow cytometry. Tumour-infiltrating CD4+ T cells showed a trending increase in combination-treated mice (figure 4*c*). Frequencies of tumour-infiltrating CD8+ T cells increased significantly, although the overall number was still low, at around 1% of CD45+ cells (figure 4*d*). Treg and immunosuppressive myeloid population frequencies were unchanged (figure 4*e*; electronic supplementary material, figure S2). Because anti-tumour T cell priming often occurs in the tumour-draining lymph node, we analysed T cells in the tumour-draining lymph nodes by IFNγ ELISpot to determine whether the modest increase in CD8+ T cells corresponded to an increase in tumour-specific T cell priming (figure 4*f*) [34,48]. After subtracting background levels of IFNγ production in wells containing no tumour cell antigen, we observed an increase in tumour-specific IFΝγ production from cells in the tumour-draining lymph node. We therefore conclude that tumour-specific, IFNγ-producing T cells were significantly increased in mice treated with radiation and checkpoint blockade, consistent with augmentation of T cell priming and activation (figure 4*f*).

## 4. Discussion and conclusion

Radiation exerts both immune-promoting and immune-suppressing effects, which complicates its use in combination immunotherapy regimens. Here, we combined dual PD-1 and CTLA-4 blockade with radiation in a poorly immunogenic model of pancreatic cancer and found that the combination therapy augments tumour-specific T cell priming. These findings are consistent with a phase II trial that evaluated two dosing regimens of SBRT combined with either durvalumab (anti-PD-L1) or durvalumab plus tremelimumab (anti-CTLA-4) [49]. In this trial, the overall response rate was 5.1% (2 partial responses of 39 evaluable). However, in a subset of patients for whom pre- and post-treatment biopsies were obtained, all patients showed an increase in intratumoural CD8+ T cells, regardless of their clinical response [49]. Collectively, these data indicate that for both mice and humans with pancreatic cancer, radiation and immune checkpoint blockade can increase T cell priming and augment anti-tumour immunity.

Mouse studies in melanoma have revealed that, when added to checkpoint blockade therapy, radiation diversifies the clonality of the anti-tumour T cell response [41]. We show a similar finding for pancreatic cancer; combination of radiation and immune checkpoint blockade can increase tumour-specific T cell priming as determined by IFNγ ELISpot. Not only do our data correspond with a T cell-mediated mechanism of tumour control, but they also generally corroborate data indicating that combining radiation with anti-PD-L1 or with checkpoint blockade and anti-CD40 therapy slows tumour growth in a KPC model of pancreatic cancer [43,44,50]. Other attempts at T cell priming using peptide-based vaccines or other T cell-directed therapies that do not induce tumour cell death have been less effective, suggesting that release of tumour antigens from dying malignant cells is a critical component of successful immunotherapy in PDAC [23,51].

Beyond increasing T cell priming, radiation-induced immunogenic cell death also induces negative feedback in the form of immunosuppressive myeloid cells that infiltrate the area to support wound healing and tissue homeostasis. Damaged tissue can not only recruit myeloid cells from circulation, but it can also affect hematopoiesis in the bone marrow, leading to the systemic release of immune-suppressive myeloid cells into circulation [52]. Previous analysis of radiation in similar models of pancreatic cancer revealed increased CCL2 in irradiated tumours leading to increased intratumoural monocytes [47]. Although we did not observe increased monocytes in our analysis of non-irradiated tumours, we did observe significant increases in SiglecF+ neutrophils, a cell type that increases in mice-bearing Kras+ tumours and is potently immunosuppressive [53,54]. The presence of this immunosuppressive population underlines the 6694c2 line's lack of immunogenicity. Functional redundancy among myeloid cells is common, as has been frequently observed in studies aimed at depleting one type of myeloid cell only to find compensatory increases in other myeloid cell types [55,56]. Notably, our addition of combination immune checkpoint blockade to radiation prevented the expansion of SiglecF+ neutrophils without generating a compensatory increase in other immune-suppressive cells, suggesting that T cell activation may be able to induce durable reprogramming of the tumour microenvironment.

PDAC is among the most treatment-refractory of all tumour types. Although induction of tumour-specific T cell responses is insufficient to provide long-term tumour control in this disease [49], we propose that the combination of radiation and immune checkpoint blockade be considered as part of combination therapy strategies.

## Data accessibility

All data are reported in the article. Raw data are available from the authors upon reasonable request.
